# Direct effects dominate responses to climate perturbations in grassland plant communities

**DOI:** 10.1038/ncomms11766

**Published:** 2016-06-08

**Authors:** Chengjin Chu, Andrew R. Kleinhesselink, Kris M. Havstad, Mitchel P. McClaran, Debra P. Peters, Lance T. Vermeire, Haiyan Wei, Peter B. Adler

**Affiliations:** 1 SYSU-Alberta Joint Lab for Biodiversity Conservation, State Key Laboratory of Biocontrol and School of Life Sciences, Sun Yat-sen University, Building No. 408, No. 135, Xingangxi Road, Guangzhou 510275, China; 2 Department of Wildland Resources and the Ecology Center, Utah State University, Logan, Utah 84322, USA; 3 USDA, ARS, Jornada Experimental Range and Jornada Basin Long Term Ecological Research, New Mexico State University, Las Cruces, New Mexico 88003, USA; 4 School of Natural Resources and the Environment, University of Arizona, Tucson, Arizona 85721, USA; 5 USDA-ARS, Fort Keogh Livestock and Range Research Laboratory, Miles City, Montana 59301, USA

## Abstract

Theory predicts that strong indirect effects of environmental change will impact communities when niche differences between competitors are small and variation in the direct effects experienced by competitors is large, but empirical tests are lacking. Here we estimate negative frequency dependence, a proxy for niche differences, and quantify the direct and indirect effects of climate change on each species. Consistent with theory, in four of five communities indirect effects are strongest for species showing weak negative frequency dependence. Indirect effects are also stronger in communities where there is greater variation in direct effects. Overall responses to climate perturbations are driven primarily by direct effects, suggesting that single species models may be adequate for forecasting the impacts of climate change in these communities.

## Abstract (web summary)

Environmental change can have indirect effects on populations by altering the outcome of competitive interactions. Here, Chu *et al*. show that although direct effects dominate the responses of grassland species to climate perturbations, indirect effects could be greater among species with smaller niche differences.

## Graphical abstract

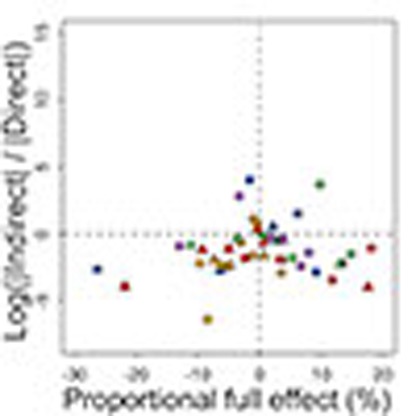

Anticipating the consequences of rapid environmental change for population and community dynamics has become an urgent challenge^1^,^2^. Meeting this challenge will require an understanding of both the direct and indirect effects of climate change. Direct effects refer to effects on the performance or abundance of a given species, assuming no changes in that species' interactions with other species. In contrast, indirect effects are mediated by interactions with other species in the community. Indirect effects can be driven either by changes in the abundance of other species or by changes in the direction and/or strength of per capita interaction effects^3^,^4^. We can define these effects of environmental change operationally: the full effect, which is what we observe in nature, is the change in the size of the focal population that is realized when other species' abundances and interactions also respond to the environmental change; the direct effect is the change in the focal species' population size that occurs if other species' abundances and interaction effects are held constant; and the indirect effect is the difference between the full and direct effects^5^.

Indirect effects are a source of uncertainty in attempts to predict the responses of communities to climate change^2^,^4^,^6^. Many studies have reported that indirect effects of climate change can amplify, outweigh or even reverse direct effects^3^,^7^. Given the potential importance of indirect effects, ignoring biotic interactions could severely affect the accuracy of forecasts of species abundances and distributions under a changing climate^2^, and consequently limit the effectiveness of conservation and management actions.

Although current research has highlighted cases where indirect effects are important, it is unknown how often this will be the case and how much of a difference indirect effects will make for ecological forecasts. Theory can help identify situations in which indirect effects are likely to be relatively weak and much simpler single-species models could be sufficient for accurate prediction. Niche differences weaken interspecific interactions, and the stronger the niche differences among species, the weaker the expected indirect effects of an environmental change^5^,^6^,^8^,^9^,^10^. Consider the extreme case of two species, A and B, which occupy completely distinct niches in time or space: the dynamics of one species will be independent of the other. In this case, climate change would not exert indirect effects. On the other hand, if A and B have overlapping niches and interact intensely, indirect effects of climate change could be strong. However, niche differences are not the only factor determining the magnitude of indirect effects.

Kleinhesselink and Adler^5^ demonstrated that for pairs of species competing for resources, the magnitude of indirect effects also increases with difference in species' direct responses to the change in the environment. This difference in environmental response is equivalent to the difference in the sensitivity of each species' density to an environmental change when growing in monocultures. When species respond in very different ways to the environment, large indirect effects can occur because species' abundances and, therefore, the relative strength of interspecific competition relative to intraspecific competition, will change greatly^5^. In contrast, if different species respond identically to the environment, there will be no change in their relative abundances, no change in the relative strength of intra- and interspecific competition, and therefore no indirect effects. This theory assumes that indirect effects are caused only by changes in competitors' relative abundance, not by changes in the per-capita competitive effects. To apply this theory, we need empirical estimates of direct and indirect effects of environmental change for many species in multiple communities, data which until now were not available.

In a previous study of the relationship between niche differences and indirect effects, Adler *et al*.^6^ applied multispecies population models to quantify direct and indirect effects, and used negative frequency dependence as the proxy for niche differences. Niche differences, such as resource partitioning or species-specific natural enemies, cause individuals to limit conspecifics more than heterospecifics. As a result, each species is more sensitive to changes in the density of conspecifics than heterospecifics. In the presence of niche differences, species' per capita growth rates decline as their relative abundance, or frequency, in a community increases, because at higher frequency individuals interact relatively more with conspecifics^11^. Therefore, the stronger the niche differentiation, the stronger the negative frequency dependence. Consistent with theory, results for a single community showed that the magnitude of indirect effects was lowest for species experiencing the strongest negative frequency dependence^6^. However, whether that result is general across multiple communities that span a wider range of negative frequency dependence remains unknown. Furthermore, Adler *et al*.^6^ did not consider how variation in direct effects might determine the strength of indirect effects. Based on Kleinhesselink and Adler^5^, we should expect stronger indirect effects in communities with greater variation in species' direct responses to environmental perturbations.

Species that occupy unique niches or respond to weather in similar ways as their competitors should experience weak indirect effects of climate change. For these species, forecasts that ignore indirect effects, such as single species models, might perform quite well. In this study, we address three research questions motivated by this theory. First, does the theoretical, negative relationship between niche differences and indirect effects observed in Adler *et al*.^6^ hold in other plant communities? Second, is the strength of indirect effects greater when there is greater variation in direct effects of climate perturbations on competitors? Third, how well can we predict species' responses to climate perturbations if we ignore indirect effects? We compiled long-term plant demographic data sets collected in the last century in five semi-arid grassland communities geographically distributed across western North America. We constructed multispecies population models to first quantify the strength of negative frequency dependence and then estimate the magnitude of full, direct and indirect effects. The results demonstrate that indirect effects are strongest for species showing weak negative frequency dependence in four of five communities and indirect effects are stronger in communities where there is greater variation in direct effects. Finally, overall responses to climate perturbations are driven primarily by direct effects.

## Results

### Climate effects on demography and cover

**Figure 1:**
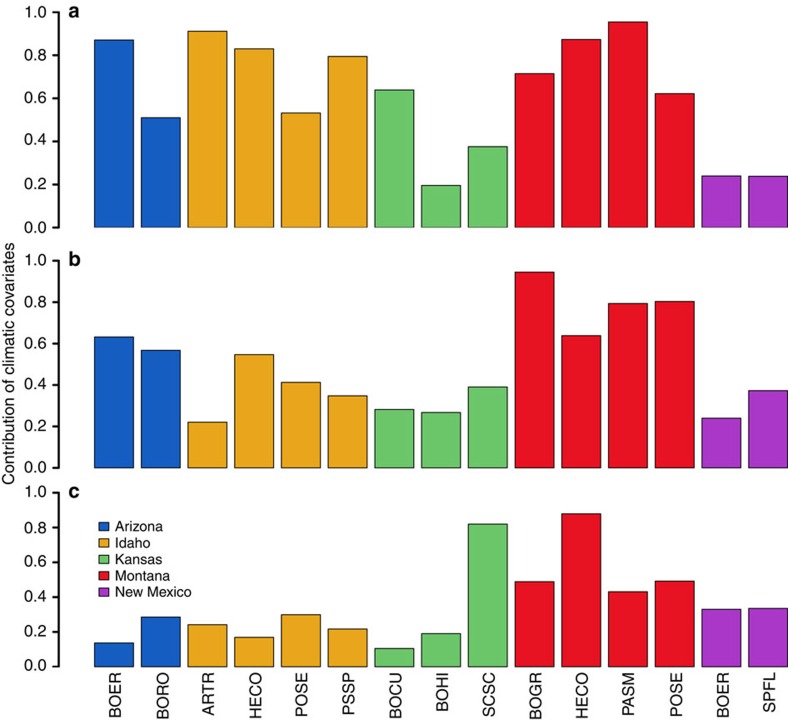
Climate covariates and variability in vital rates. The proportion of interannual variability in vital rates explained by climatic covariates for: (**a**) survival, (**b**) growth and (**c**) recruitment.

For the 12 species we analysed, the inclusion of climatic covariates explained from 22 to 94% of the interannual variation in growth rates, with a mean of 50% (Fig. 1 and Supplementary Table 1). For survival, the contribution of climatic covariates ranged from 8.3 to 96%, with a mean of 59%. For recruitment, the climatic covariates were relatively less effective, reducing the residual deviance associated with interannual variability by 10–88%, with a mean of 36%.

**Figure 2:**
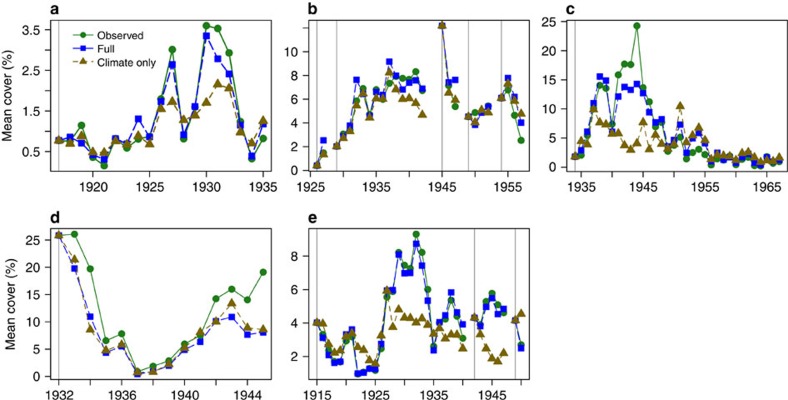
Comparison between predicted and observed abundance reveals the ecological importance of climatic covariates. Comparison of observed (circles) and predicted mean cover from simulations with an IBM that included both climatic covariates and random year effects (squares) or climatic covariates only (triangles). One species from each study site is presented here: (**a**) *B. rothrockii* in Arizona, (**b**) *A. tripartita* in Idaho, (**c**) *B. curtipendula* in Kansas, (**d**) *B. gracilis* in Montana and (**e**) *B. eriopoda* in New Mexico. Results for the remaining species are shown in Supplementary Fig. 1.

A second approach for evaluating the ecological importance of climatic covariates relied on individual-based model (IBM) simulations that compared predicted and observed cover in the historical plots (Fig. 2, Supplementary Fig. 1 and Supplementary Table 2). For IBM models with climatic variables only, the correlation coefficients between the predicted cover and observed cover across years ranged from 0.22 to 0.99 (with a mean of 0.79). If both random year effects and climatic covariates were included in the models, the correlation coefficients ranged from 0.83 to 0.99 (with a mean of 0.95).

### Negative frequency dependence and indirect effects

**Figure 3:**
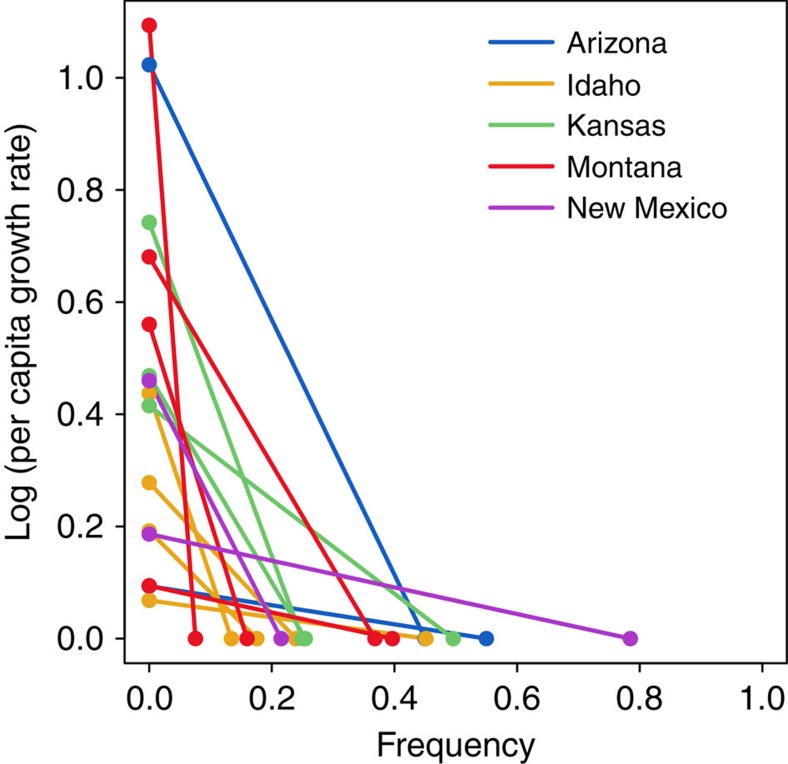
Negative frequency dependence. The estimated relationship between per capita growth rate of each species and its equilibrium frequency. The slope of the line represents the magnitude of the negative frequency dependence for each species. The per capita growth rate is 1 (zero when log transformed) when a species is at its equilibrium frequency.

All species showed positive invasion growth rates and negative frequency-dependent patterns of population growth, that is, a negative relationship between a species' per capita growth rate (on the log scale) and its frequency, or relative cover, in the community (Fig. 3 and Supplementary Table 3). *Pascopyrum smithii* in Montana exhibited the strongest negative frequency dependence with a slope of −14.46, whereas *Artemisia tripartita* in Idaho had the weakest negative frequency dependence with a slope of −0.15. The mean negative frequency dependence for all species was −2.41. We used these measures of negative frequency dependence as a proxy measure of niche differences between each species and its competitors.

Species cover showed idiosyncratic responses to the climate perturbations, with full effects of the perturbations varying in both strength and direction (Supplementary Fig. 2). For example, a 1% precipitation increase decreased the abundance of *A. tripartita* and *Pseudoroegneria spicata*, but increased the abundance of *Hesperostipa comata* and *Poa secunda* in Idaho.

**Figure 4:**
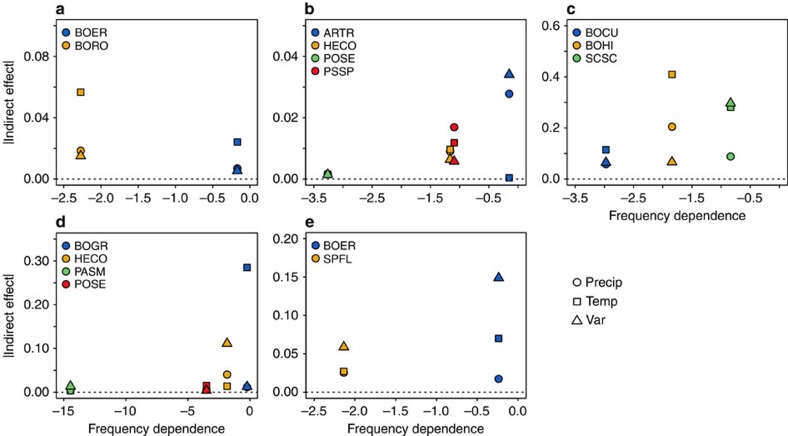
Negative frequency dependence and indirect effects of climatic covariates. The relationship between the slope of negative frequency dependence and the absolute values of indirect effects of precipitation (‘Precip'), temperature (‘Temp') and variability (‘Var') perturbations on change in cover for each species. (**a**–**e**) Sites in Arizona, Idaho, Kansas, Montana and New Mexico, respectively.

In four out of five communities, the strength of indirect effects decreased with stronger negative frequency dependence (Fig. 4). For instance, *A. tripartita* in Idaho and *Bouteloua gracilis* in Montana experienced the weakest negative frequency dependence and the strongest indirect effects of the climate perturbations of any species in their respective communities. A linear mixed-effects model including all species and all sites showed that the magnitude of raw indirect effects declined significantly with negative frequency dependence (mixed-effects model: coefficient=0.0067, *P*=0.039, DF=29 and *t*-value=2.162; Fig. 4 and Table 1). The Arizona community was the exception (Fig. 4): *Bouteloua rothrockii* had stronger negative frequency dependence than *Bouteloua eriopoda* but experienced larger indirect effects.

### Variation in direct effects influences indirect effects

The direct effect of precipitation on equilibrium cover was positive for all species except two, *A. tripartita* in Idaho and *P. secunda* in Montana (Supplementary Fig. 2). In contrast, the indirect effects of precipitation mediated by competition were usually negative, with the exception of *B. eriopoda* in Arizona and *Bouteloua curtipendula* in Kansas. Variability in temperature and precipitation drove positive direct effects for seven species and negative direct effects for the others. The indirect effects of variability were usually negative, except for *B. rothrockii* in Arizona, *A. tripartita* in Idaho, and *H. comata* and *P. smithii* in Montana. The direct and indirect effects of temperature were more idiosyncratic across species (Supplementary Fig. 2).

**Figure 5:**
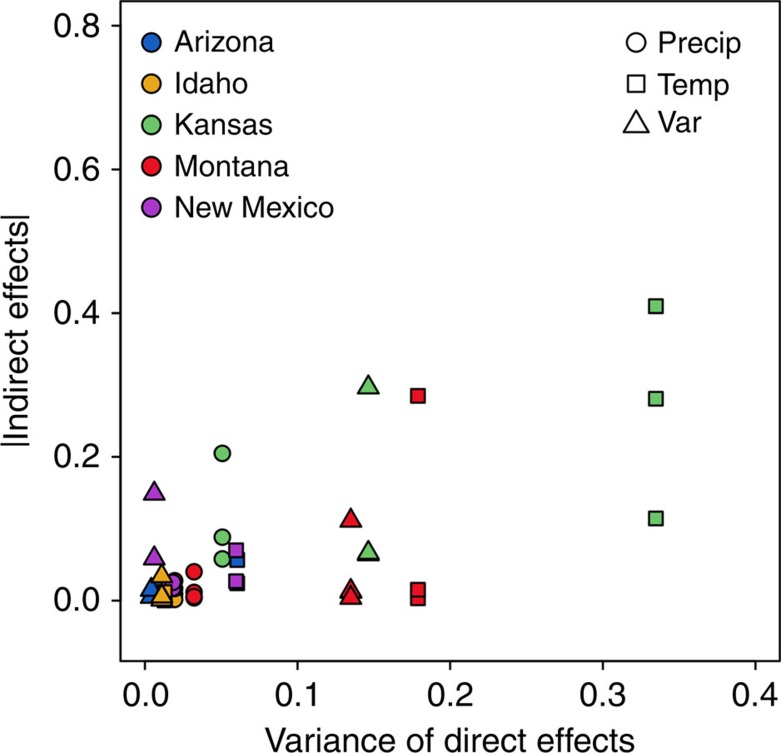
Variation in direct effects and indirect effects of climatic covariates. The relationship between community-level variances of direct effects and the absolute values of indirect effects of precipitation (‘Precip'), temperature (‘Temp') and variability (‘Var') perturbations on change in cover for each species.

The linear mixed-effects model showed that the strength of raw indirect effects significantly increased with the community-wide variation in direct effects of climate perturbations (mixed-effects model: coefficient=0.6007, *P*=0.0013, DF=9 and *t*-value=4.594; Fig. 5 and Table 1).

### Relative importance of indirect and direct effects

**Figure 6:**
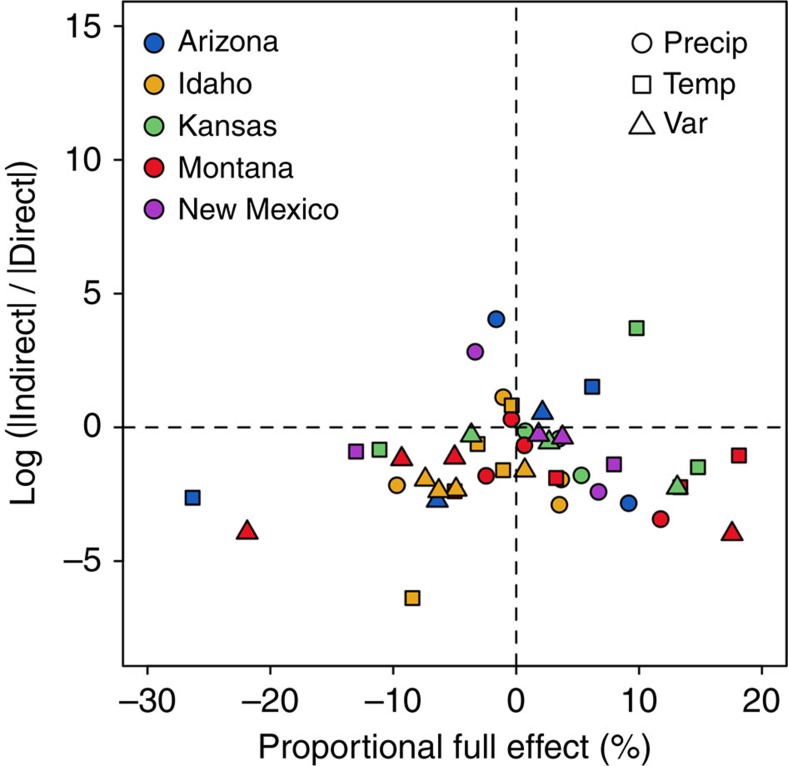
Relative importance of direct and indirect effects of climatic covariates. The relationship between the proportional full effects and the log ratios of absolute indirect and direct effects on change in cover for each species. Positive values of log ratios indicate that indirect effects are stronger than direct effects.

Log ratios between proportional indirect effects (raw indirect effects scaled by species equilibrium abundances) and proportional direct effects were <0 in 37 of 45 climate perturbation scenarios (Fig. 6), indicating that indirect effects were weaker than direct effects. For the eight cases in which the log ratios were >0, the proportional full effects were close to 0, with the exceptions of *Bouteloua hirsuta* in Kansas (9.8% proportional change) and *B. rothrockii* in Arizona (6.1%) under the temperature perturbation, and *Sporobolus flexuosus* in New Mexico (−3.3%) under the precipitation perturbation (Fig. 6). In addition, the proportional full effects were tightly correlated with the proportional direct effects (*r*=0.94) (Supplementary Fig. 3). However, the relationship between the proportional full effects and the proportional indirect effects was weak (*r*=0.13) (Supplementary Fig. 3).

## Discussion

Theory predicts that the magnitude of indirect effects of climate change should decrease with increasing niche differences^5^,^6^ and increase with variation in species' direct responses to climate change^5^. Our results support both of these predictions.

In four of the five communities we studied, which includes the sagebrush steppe community that we previously analysed^6^, species experiencing stronger negative frequency dependence, a proxy for niche differences, were less sensitive to the indirect effects of climate change (Fig. 4). Furthermore, the overall nonlinear shape of the relationship, with the maximum strength of raw indirect effects decreasing rapidly as negative frequency dependence declines from zero, is consistent with theory^5^. However, we also found great variation in the strength of indirect effects (Figs 4 and 5). For species that experienced weak negative frequency dependence (>−2.0 in Fig. 4), indirect effects of different climate perturbations could be very strong or very weak, whereas for species experiencing strong negative frequency dependence (<−3.0 in Fig. 4), indirect effects were uniformly weak.

The reason that species with weak negative frequency dependence display such wide variation in indirect effect strength is that niche differences are not the only factor influencing the magnitude of indirect effects. The size of indirect effects also depends on variation in direct effects experienced by the species in the community (Fig. 5 and Table 1). For instance, in Kansas and Montana variability in direct effects of temperature was high; some species responded positively, whereas others responded negatively to the same change in temperature (Supplementary Fig. 2). As a result, these communities showed the strongest indirect effects of temperature perturbations (Fig. 4). In fact, if we did not account for the variance in direct effects, our linear mixed-effects model could not detect a significant impact of negative frequency dependence (mixed-effects model; coefficient=0.0059, *P*=0.1025, DF=29 and *t*-value=1.69; Supplementary Table 4).

Another factor contributing to variability in the size of raw indirect effects is asymmetry in interspecific interactions^5^. The Arizona community was the only one in which the relationship between indirect effects and negative frequency dependence did not match our prediction: *B. eriopoda* had weaker negative frequency dependence than *B. rothrockii*, but it was less sensitive to the indirect effects of climate perturbations (Fig. 4). This outcome occurred because *B. eriopoda* always experienced stronger direct effects than *B. rothrockii* (Supplementary Fig. 2) and it had strong effects on *B. rothrockii* vital rates, whereas *B. rothrockii* had little effect on the performance of *B. eriopoda* (see Supplementary Data 1,2,3; coefficients for the effect of *B. eriopoda* on *B. rothrockii* survival, growth and recruitment rates were −0.017, −0.0055 and −0.61, respectively; in contrast, coefficients for the effect of *B. rothrockii* on *B. eriopoda* for these three vital rates were 0.0, 0.0 and −0.28). These asymmetries overwhelmed the stabilizing effect of negative frequency dependence on *B. rothrockii*. In the other four communities, the theoretical relationship between niche differences and indirect effects emerged despite the noise introduced by such idiosyncrasies.

An implication of the negative relationship between niche differences and the absolute magnitude of indirect effects is that single-species models, which treat plant–plant interactions implicitly, might be appropriate for predicting climate change impacts on species occupying unique niches. We found that for most species and climate perturbations the indirect effects were weaker than the direct effects (Fig. 6). In addition, the full effects of climate perturbations primarily reflected direct effects: proportional full effects were strongly correlated with proportional direct effects (*r*=0.94), but not with proportional indirect effects (*r*=0.13) (Supplementary Fig. 3). In other words, single-species models would work well for most of our species, not just those experiencing strong negative frequency dependence. This result is consistent with a separate analysis we conducted on the same data sets showing evidence for very strong niche differences^12^. Because of the large niche differences among the species we modelled, species interactions and, in turn, indirect effects are weak. However, 8 of the 45 climate perturbation scenarios did show some degree of indirect effects (Fig. 6), which suggests that the model structure we chose to fit empirical data does not prevent the detection of indirect effects.

We found no general patterns in the net effects of particular climate perturbations on our study species (Supplementary Fig. 2). Precipitation had positive full effects on 8 of 15 cases, but negative full effects on others. Temperature increased equilibrium cover for seven species but decreased the cover for others. Similarly, the variability of precipitation and temperature positively influenced the equilibrium cover for seven species but negatively influenced others. These results illustrate the diversity of species' responses to climate variation.

Our conclusions are tempered by several caveats. First, our estimates of the strength of niche differences are based on observational data. Estimating the strength of density dependence in observational data is notoriously difficult^13^. Although we have worked hard to rule out biases resulting from measurement error^12^, the simulations we ran to estimate negative frequency dependence may extrapolate beyond the range of observed interaction neighbourhoods used to fit the models. Although overestimates of niche differences would not affect our test of the theoretical relationship between niche differences and indirect effects, they could affect our conclusions about the relative importance of direct and indirect effects. In addition, our results are site specific: the same species might experience greater niche overlap at a site with different climate, soil conditions or community composition. The relative importance of indirect effects to direct effects could change across a species' range. For instance, *H. comata* in Idaho and Montana responded in a similar way to the variability perturbation, but not to the precipitation and temperature perturbations.

Second, as sample-size requirements limited our analysis to common, co-occurring species, we can only speculate about the sensitivity of less common species to indirect effects. Some species may be rare due to strong competitive suppression from dominants; for these species, we would expect smaller niche differences and stronger indirect effects in comparison with our results for common species. However, species that are both persistent and rare may be relatively insensitive to interspecific competition. In fact, theory suggests rare species might actually experience stronger niche differences than common species^14^. In this case, rare species might experience even weaker indirect effects than those we observed for our common study species. Testing this conjecture would be especially fascinating in more species-rich communities. For example, our approach for estimating direct and indirect effects of climate variation could be applied to long-term observational data from tropical forests ecosystems. If niche differences in hyperdiverse communities are smaller than those we observed here, the indirect effects of climate change should be larger.

Third, our analysis assumed that climate perturbations would not alter interaction coefficients. In our simulations, we held the interaction coefficients constant, so that indirect effects of climate perturbations were mediated only by changes in species abundances. Changes in the per capita interaction coefficients would be especially important if they alter niche differences, making a species more or less sensitive to indirect effects. However, evidence for environment-driven changes in the magnitude or direction of per capita interaction effects remains mixed^15^,^16^. Furthermore, given the strength of niche differences that we observed, the interaction coefficients would have to change dramatically, to substantially weaken niche differences and strengthen indirect effects. More probable causes of altered niche differences include adaptive responses to directional climate change or colonization by novel competitors better adapted to the changing climate^17^, which our analysis does not consider.

Finally, our study focused only on plant–plant interactions, but species responses to climate change will also be influenced by trophic interactions. Studies indicate that trophic interactions may generate strong indirect effects of climate change^18^. Although our study does not explicitly address trophic interactions, it does show how theory can be used to predict variation in the strength of species interactions and the resulting indirect effects.

In four of the five communities we studied, the magnitude of indirect effects of climate perturbations were largest for species experiencing weak negative frequency dependence and decreased rapidly for species with increasingly negative frequency dependence, consistent with theory. This relationship emerged despite considerable variation in the strength and direction of indirect effects caused by idiosyncratic relationships between climate drivers and species performance. Our results also revealed stronger indirect effects in communities with more variation among species in the strength and direction of direct effects. Overall, indirect effects were weaker than the direct effects of climate perturbations. Although this result implies that single-species models might be appropriate for forecasting the response of co-occurring species to climate change, we did not consider trophic interactions or the eventual influence of novel competitors. Our work demonstrates how coexistence theory can inform application-driven global change research, but a significant challenge is acquiring the long-term demographic data needed to estimate niche overlap.

## Methods

### Roadmap of the analysis

Our approach involved five steps: (1) extracting demographic rates from yearly mapped quadrats; (2) fitting statistical models for growth, survival and recruitment; (3) constructing two types of multiple-species dynamic models, IBMs and integral projection models (IPMs); (4) simulating the models to quantify negative frequency dependence; and (5) performing simulations with additional climate perturbations to disentangle direct from indirect effects. Although our analyses depend on models, the models are empirical—they were fit directly to observed data.

### Data set description

In the early twentieth century, scientists at numerous experiment stations in semi-arid western US grasslands began mapping permanently located quadrats and continued annual censuses for decades. Here we focused on five long-term ‘chart quadrat' data sets from Sonoran desert in Arizona, sagebrush steppe in Idaho, southern mixed prairie in Kansas, northern mixed prairie in Montana and Chihuahuan desert in New Mexico^19^,^20^,^21^,^22^. Hereafter, we use the state name to refer to each plant community (Table 2). All individual perennial plants within each 1-m^2^ quadrat were identified and mapped yearly using a pantograph^23^. Mapped polygons represented the basal cover of individual grasses and canopy cover of individual shrubs.

To select the quadrats and species for the present analyses (Table 2), we visually inspected all maps for each study site and checked for completeness and accuracy. Next, as fitting our models requires large samples sizes, we identified species with relatively high frequency across quadrats within each site (above 20%). We then performed a non-metric multidimensional scaling ordination to determine which quadrats shared a similar composition of these common, co-occurring species. Based on the degree of aggregation of quadrats on the non-metric multidimensional scaling plot, we selected corresponding quadrats and species for our analyses. In addition, we confirmed our selections with scientists familiar with each study site. This process resulted in the selection of 12 target species with three species occurring in two study sites (Table 2).

Monthly precipitation and temperature records were available for each study site, except for the Arizona site^19^,^20^,^21^,^22^, for which we extracted precipitation and temperature data from PRISM (http://www.prism.oregonstate.edu/). Based on the timing of the growing season at each site and previous analyses^6^,^24^, we chose three precipitation covariates and two temperature covariates a priori (Supplementary Table 5): the annual or water-year precipitation in the year preceding an observed year-to-year transition, the precipitation and temperature of the critical seasons in the first year of a transition and the precipitation and temperature of the critical seasons in the second year of a transition, respectively. The mean temperature across the critical seasons was used for all sites except Kansas, where maximum temperature was analysed (a model based on mean temperature simulated unrealistically high cover for *Schizachyrium scoparium* compared with its observed cover). We also considered interactions between precipitation and temperature in each year in the statistical models.

### Extracting demographic data from mapped quadrats

We applied a computer programme to track the identity of individual genets based on their spatial locations in the permanent quadrats^25^,^26^. To take into account mapping error and the potential for herbaceous perennials to ‘move' short distances via resprouting, the first step in the tracking algorithm was to add a 5-cm buffer around each mapped polygon^25^,^26^,^27^. After adding the buffer area to all polygons of each focal species in year *t*−1, we then calculated the overlap of each of these polygons with each conspecific polygon occurring in year *t*. If the year *t* polygon did not overlap with any conspecific polygon from the previous year, it was labelled as a new recruit. Otherwise, the individual was considered a survivor and inherited the identity of the polygon with which it shared the greatest overlap area. Our approach allowed genets to fragment and/or coalesce over the study period.

To parameterize our models, we chose to represent each genet in each year as a circle with an area equal to the sum of all the polygons that compose the genet and a location corresponding to the polygons' centroid^27^. Multiple genets could have virtually identical centroids when the polygons that compose each genet are interspersed. Very small plants were originally mapped as points; we represented those plants as circles with an area of 0.25 cm^2^. The distance between two genets was defined as the distance between their centroids.

### Fitting statistical models of vital rates

We first describe our statistical models for survival, growth and recruitment, which extended the models of Chu and Adler^12^ to include climate covariates. We then explain how we quantified the interannual variation in vital rates explained by the climatic covariates.

We modelled survival and growth as functions of genet size, climate covariates and interactions with neighbouring conspecific and heterospecific genets^12^. We incorporated plant–plant interactions by calculating indices of local intraspecific and interspecific crowding. We assumed that the crowding experienced by a focal genet depends on the distance, *d*, to neighbours and the size of those neighbours, *u*:


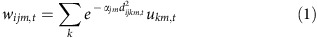


where *w*_*ijm*,*t*_ is the crowding that genet *i* of species *j* in year *t* experiences from neighbours of species *m*, *α*_*jm*_ determines the spatial scale over which neighbours of species *m* exert influence on a genet of species *j* (*m*=*j* means the intraspecific interaction), *k* indexes all the focal genet's neighbours of species *m* at time *t* and *d*_*ijkm*,*t*_ is the distance between genet *i* in species *j* and genet *k* in species *m*. The use of squared distances implies a Gaussian competition kernel. We first selected *α*_*jj*_ values for each single-species model and translated those values into *α*_*jm*_ for interspecific interactions^6^,^12^. *α*-values for each species for growth and survival were directly extracted from Chu and Adler^12^.

We modelled the survival probability, *S*, of genet *i* in species *j* and group *g* from time *t* to *t*+1 as


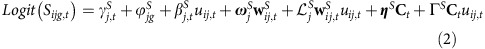


where *γ* is a time-dependent intercept, *ϕ* is the coefficient for the effect of group defined as a set of nearby quadrats located within one pasture or grazing exclosure and *β* is the coefficient that represents the effect of genet size *u* on survival, the vector of ***ω***'s contains competition effects describing the impact of crowding ***w*** by each species on the focal species *j*, the 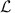
's are interactions between the impact of crowding and genet size, ***η*** are the effects of the climate covariates ***C*** on the survival intercept and Γ are the effects of interactions between the climate variables and genet size.

We modelled changes in genet size (log transformed) from time *t* to *t*+1, conditional on survival, with a similar structure:


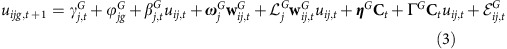


This model assumes constant variance around expected growth. To capture non-constant error variance in growth, we followed previous studies^28^,^29^,^30^ in modelling the variance *ɛ* about the growth curve (equation (3)) as a separate nonlinear function of predicted genet size:


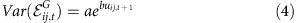


We used a stepwise variable selection procedure based on Akaike's Information Criterion to select the best combination of climatic variables from the set of three precipitation and two temperature covariates and their interactions^31^. The stepwise variable selection procedure was conducted using ‘*stepAIC*' function in R package ‘MASS'. We fitted the final models with the selected climate covariates using ‘*lmer*' (for growth) and ‘*glmer*' (for survival) in R package ‘lme4', treating year and spatial location as random effects, that is, random intercepts for year, random slopes for genet size in each year and random intercepts for group. Detailed results for the survival and growth models are presented in Supplementary Data 1 and 2.

As we could not determine which recruits were produced by which potential parent genets, we modelled recruitment at the quadrat level rather than on the individual genet level. We assumed that the number of individuals, *y*, of species *j* recruiting at time *t*+1 in the location *q* follows a negative binomial distribution:


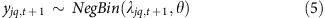


where *λ* is the mean intensity and *θ* is the size parameter. In turn, *λ* depends on the composition of the quadrat in the previous year:


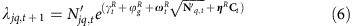


where *R* refers to recruitment, *N′*_*jq,t*_ is the ‘effective cover' (cm^2^; see below) of species *j* in quadrat *q* at time *t*, *γ* is a time-dependent intercept, *ϕ* is a coefficient for the effect of group location, ***ω*** is a vector of coefficients that determine the strength of intra- and interspecific density dependence, ***N***′_*q*,*t*_ is the vector of ‘effective cover' of each species in quadrat *q* and time *t*, and ***η*** are the effects of the climate covariates, **C**. Equation (6) is based on a Ricker equation for discrete time population growth^32^, but we used the square root of local cover in the exponential term because it improved model fit. To recognize the possibility that plants outside the mapped quadrat could contribute recruits to the focal quadrat, we estimated ‘effective cover' as a mixture of the observed cover *N*, in the focal quadrat *q* and the mean cover across the group *g* in which the quadrat was located:


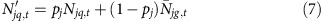


where *p* is the mixing fraction between 0 and 1.

Following previous work^6^,^12^,^29^, we treated year and group variables as random factors allowing intercepts to vary among years and spatial locations. We used the same five climate covariates that we used for survival and growth, but did not fit the interactions between precipitation and temperature as they hampered convergence. We estimated parameters in a Bayesian framework, implementing Markov Chain Monte Carlo simulations through *WinBUGS* 1.4 (ref. 33). Convergence was observed graphically for all parameters and diagnosed using the Brooks–Gelman statistic^34^. The full results for the recruitment models are presented in Supplementary Data 3.

In our models, temporal variation in vital rates is introduced by both climate covariates and random year effects. To provide some indication of the relative influence on vital rates of our selected climate covariates, we quantified the portion of interannual variation in vital rates explained by the climate covariates. We fit (i) a ‘constant' model with no year effects or climate covariates (that is, no temporal variation); (ii) a ‘climate' model only including climate covariates; and (iii) a ‘full' model with both random year effects and climate covariates. We then calculated the proportion of temporal variability in vital rates explained by the climate covariates as:


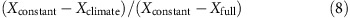


where *X* is the sum of the squared residuals (growth regressions) or the residual deviance (survival and recruitment regressions).

### Constructing multispecies population models

We used the parameterized vital rate regressions to construct two multispecies dynamic population models: IBMs and IPMs. We used IBMs to evaluate the models' ability to reproduce the observed dynamics and to quantify the influence of climatic covariates on vital rates. We used IPMs to estimate negative frequency dependence and the direct and indirect effects of climate perturbations.

Simulations of the IBM began by specifying the sizes, locations and species identities of modelled plants. At each time step, we applied the recruitment regression to determine the number of recruits to appear in the following time step and applied the survival and growth regressions to determine the fate of each individual plant. We randomly assigned spatial location for each new recruit. In these IBM simulations, demographic stochasticity arose from all three vital rates. A negative binomial distribution was used to describe the number of recruits, a Bernoulli distribution was used to describe the survival of each genet and changes in genet sizes were described by a normal distribution with size-dependent variance (equation (4)).

To reproduce observed dynamics, we simulated time series of abundances in each quadrat by initializing the IBM with the actual conditions (plant sizes and locations) observed in the first year the quadrat was recorded. We then projected the model forward in time, drawing from the year-specific parameters in chronological order and applying location-specific random effects as appropriate for each quadrat. For these simulations we used absorbing boundaries. Where data gaps in the observed time series occurred, we re-initialized the IBM with the plant sizes and locations observed in the year following the gap. For each quadrat, we ran 50 replicate simulation runs and averaged cover and density across runs for each species.

The demographic stochasticity present in the IBM complicated the analyses of asymptotic behaviour, such as long-term population growth rates and equilibrium abundances. Therefore, we built an environmentally stochastic, but demographically deterministic, version of the IBM using an IPM approach. IPMs provide an elegant way to model the dynamics of continuously (size) structured populations in discrete time^28^,^30^.

In our IPM, the population of species *j* is represented by a density function *n*(*u*_*j*_, *t*), which gives the density of genets of size *u* at time *t*, with genet size on natural-log scale, that is, *n*(*u*_*j*_, *t*)*du* is the number of genets whose area (on arithmetic scale) is between exp(*u*_*j*_) and exp(*u*_*j*_+*du*). The density function for size *v* at time *t*+1 is given by


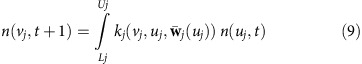


where the kernel 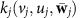 describes all possible transitions from size *u* to *v* and 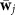 is a vector whose elements are the average crowding experienced by an individual of size *u*_*j*_ and species *j* from each of the other species. We describe below how 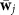 is calculated. The integral is evaluated over all possible sizes from a lower size limit *L* to an upper size limit *U* that extends past the range of observed sizes.

The kernel is constructed from the fitted survival (*S*), growth (*G*) and recruitment (*R*) models:


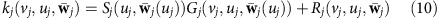


*S* is given by equation (2) and *G* by equation (3). The recruitment function *R*, defined in equations (5) and (6), gives the number of new recruits produced per quadrat. To incorporate this recruitment function into the IPM, we assumed that fecundity increases linearly with size, 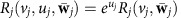
29,35; this has the consequence that recruitment by any species in the IPM is proportional to the species' total cover, as desired.

In the vital rate regressions and the IBM, we calculated a neighbourhood crowding *w*_*ij*_ unique to each individual *i* based on the spatial locations and sizes of neighbouring plants (equation (1)). As the IPM is not spatially explicit, we developed a spatially implicit approximation to 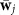 that captures the essential features of neighbourhood competition. We found that in the observed data and IBM simulations, heterospecific individuals were randomly distributed, but conspecific individuals displayed non-random, size-dependent spatial patterns. Specifically, although small genets were randomly distributed, large genets were segregated from each other without overlapping in area^6^,^12^,^29^. Our approximation for neighbourhood crowding distinguishes between intraspecific and interspecific neighbours, applying a conspecific ‘no overlap' rule^12^,^29^.

### Quantifying negative frequency dependence

Negative frequency dependence describes the relationship between a species' per capita growth rate and its frequency in the community^6^,^11^. In contrast to the pairwise niche difference defined by Chesson^8^, negative frequency dependence provides one metric that represents the niche difference between a focal species and the rest of the community^6^. To quantify the degree of negative frequency dependence experienced by each species, we estimated each species' equilibrium frequency and invasion growth rate through two different simulations of the IPM. To estimate equilibrium frequency, we initialized the IPM with very low abundances and ran the model long enough to reach a stochastic equilibrium, randomly drawing from the observed climate covariates and random year effects at each time step, then recorded the equilibrium cover for all species. To obtain the invasion growth rate of a focal species, we allowed the other species in the community to reach their equilibrium abundances, while holding the abundance of the focal species low enough so that it could not influence others or itself^8^. We began by simulating a community containing all species, except the focal species, and ran the simulation until the community reached a stochastic equilibrium. Next we introduced the focal species at very low abundance (a cover of 10^−6^). The invasion growth rate was calculated as log(*C*_*t+1*_*/C*_*t*_), where *C* is the cover of the focal species. After recording the change in cover, we returned the cover of the focal species to 10^−6^ and repeated the one-step invasion experiment. At each time step, we randomly chose climate covariates from one observed year and, independently, one set of random year effects. The long-term invasion growth rate of the focal species was estimated as the species' (geometric) mean growth rate over the period of 1,000 time steps. We repeated these steps for each target species in each community. We quantified negative frequency dependence as the slope of the line linking the equilibrium frequency and the invasion growth rate.

### Disentangling direct and indirect effects of perturbations

Detecting indirect effects of climate perturbation is challenging, because species' observed responses represent the net outcome of direct and indirect effects^6^,^36^. More formally, this ‘full effect' of a climate perturbation is the sum of the ‘direct effect' and the ‘indirect effect'. Therefore, if we can estimate the full effect and direct effect, we can readily obtain the ‘indirect effect'.

To quantify the ‘full effect' of climate change, we perturbed the IPM by increasing the means of the observed precipitation or temperature covariates, or by increasing the variability in precipitation and temperature, allowing all species in a given community to respond simultaneously. Our choice of perturbations reflected the expectation that elevated concentrations of greenhouse gases in the atmosphere will alter both the means and variances of climatic covariates. We modified the means of climatic covariates with a relatively small perturbation (1%) to avoid questions about extrapolating our models well beyond the range of historical variation. As our perturbations of variances had weaker effects, we increased variance by 10% to facilitate comparisons with our mean perturbations. We defined species' baseline as the equilibrium cover from IPM simulations based on observed climate covariates. Next we calculated the ‘full effect' of each climate perturbation by comparing baseline cover with the equilibrium cover of each species from simulations based on perturbed climate.

To simulate direct effects of the climate perturbations, we focused on one species at a time. In each simulation, vital rates of the focal species were determined by the perturbed climate variable(s), but the vital rates of the other species were determined by the unperturbed observed climate variable(s). We conducted these two parallel simulations with the same sequence of randomly generated climate years and random effect years. The difference of the equilibrium cover of the focal species and the corresponding baseline cover represents the ‘direct effect' of climate perturbation on this focal species. The indirect effect for each species was the difference between the full and direct effects. To compare the relative importance of direct and indirect effects across species that differ in total cover, we rescaled the ‘raw' effects by dividing by each species' equilibrium cover. We referred to these rescaled effects as ‘proportional' full, direct and indirect effects. We calculated log ratios between absolute proportional indirect effects and absolute proportional direct effects, with positive values indicating that indirect effects were stronger than direct effects.

To identify the factors generating variation in indirect effects of climate perturbations, we constructed a linear mixed-effects model in which the absolute magnitude of raw indirect effects was the response variable and the fixed factors were negative frequency dependence and the community-level variance of raw direct effects. For each climate perturbation, all species at one study site had the same value of variance in raw direct effects. The random effects were study site and climate perturbation nested within study site. We fitted the model using ‘*lme*' in R package ‘nlme.'

### Data availability

Data and computer code used to fit the vital rates models and to run simulations have been deposited in Dryad: doi:10.5061/dryad.f1860.

## Additional information

**How to cite this article:** Chu, C. *et al*. Direct effects dominate responses to climate perturbations in grassland plant communities. *Nat. Commun.* 7:11766 doi: 10.1038/ncomms11766 (2016).

## Supplementary Material

Supplementary Information Supplementary Figures 1-3, Supplementary Tables 1-5

Supplementary Data 1 Statistical results for the survival models of species.

Supplementary Data 2 Statistical results for the growth models of species.

Supplementary Data 3 Statistical results for the recruitment models of species.

## Figures and Tables

**Table 1:** Results for the mixed-effects model that explains variation in the indirect effects of climate perturbations.

Variable	Estimated value	s.e.	DF	*t*-value	*P*-value
(Intercept)	0.0320	0.01940	29	1.649	0.1099
NFD	0.0067	0.00310	29	2.162	0.0390
Variance in direct effects	0.6007	0.13077	9	4.594	0.0013

NFD, negative frequency dependence.

NFD and the variance in direct effects were treated as fixed factors, whereas study site (*n*=5) and climate perturbation nested within study site (*n*=15) were treated as random effects. The number of total observations was 45. The linear mixed-effects model was fit by maximum likelihood with the function ‘*lme*' in R package ‘nlme.'

**Table 2:** Information on the five chart quadrat data sets.

Location	Santa Rita Exp. Range, AZ	US Sheep Exp. Station, ID	Hays, KS	Fort Keogh, MT	Jornada Exp. Range, NM
Vegetation	SD	SBS	SMP	NMP	CD
Elevation (m)	1,150	1,650	650	720	1,260
Lat/Long	31.83/ −110.88	44.2/ −112.1	38.8/ −99.3	46.32/ −105.8	32.62/−106.67
Precip/Temp.	350 mm/16 °C	325 mm/6 °C	585 mm/13 °C	343 mm/8 °C	246 mm/14 °C
Precip.season	Summer	Fall-Spring	Spr-Summer	Spr-Summer	Summer-Fall
Quadrats	178/22	26/26	51/6	44/19	70/40
Yearly census period	1915–1935	1923–1957	1932–1972	1932–1945	1915–1950
Species selected	*B. eriopoda B. rothrockii*	*A. tripartita Hesperostipa comata P. secunda P. spicata*	*B. curtipendula B. hirsuta S. scoparium*	*B. gracilis H. comata P. smithii P. secunda*	*B. eriopoda S. flexuosus*
Species code	**BOER**, BORO	ARTR, **HECO**, **POSE**, PSSP	BOCU, BOHI, SCSC	BOGR, **HECO**, PASM, **POSE**	**BOER**, SPFL

CD, Chihuahuan desert; NMP, northern mixed prairie; SBS, sagebrush steppe; SD, Sonoran desert; SMP, southern mixed prairie.

The row ‘Quadrats' shows the total number of quadrats mapped (*x*) in each ecosystem, relative to the number of quadrats selected (*y*) for the current analysis (*x*/*y*). Three species present in two study sites were marked with bold font in the row ‘Species code'.

## References

[b1] Thomas C. D. . Extinction risk from climate change. Nature 427, 145–148 (2004). 14712274 10.1038/nature02121

[b2] Angert A. L., LaDeau S. L. & Ostfeld R. S. Climate change and species interactions: ways forward. Ann. N. Y. Acad. Sci. 1297, 1–7 (2013). 25098378 10.1111/nyas.12286

[b3] Tylianakis J., Didham R., Bascompte J. & Wardle D. Global change and species interactions in terrestrial ecosystems. Ecol. Lett. 11, 1351–1363 (2008). 19062363 10.1111/j.1461-0248.2008.01250.x

[b4] Gilman S. E., Urban M. C., Tewksbury J., Gilchrist G. W. & Holt R. D. A framework for community interactions under climate change. Trends Ecol. Evol. 25, 325–331 (2010). 20392517 10.1016/j.tree.2010.03.002

[b5] Kleinhesselink A. R. & Adler P. B. Indirect effects of environmental change in resource competition models. Am. Nat. 186, 766–776 (2015). 26655983 10.1086/683676

[b6] Adler P. B., Dalgleish H. J. & Ellner S. P. Forecasting plant community impacts of climate variability and change: when do competitive interactions matter? J. Ecol. 100, 478–487 (2012).

[b7] Suttle K., Thomsen M. & Power M. Species interactions reverse grassland responses to changing climate. Science 315, 640–642 (2007). 17272720 10.1126/science.1136401

[b8] Chesson P. Mechanisms of maintenance of species diversity. Annu. Rev. Ecol. Syst. 31, 343–366 (2000).

[b9] Chesson P. in Encyclopedia of Sustainability Science and Technology ed. Meyers R. A. 223–256Springer-Verlag (2013).

[b10] Godsoe W., Murray R. & Plank M. J. The effect of competition on species' distributions depends on coexistence, rather than scale alone. Ecography 38, 1071–1079 (2015).

[b11] Adler P. B., HilleRisLambers J. & Levine J. M. A niche for neutrality. Ecol. Lett. 10, 95–104 (2007). 17257097 10.1111/j.1461-0248.2006.00996.x

[b12] Chu C. J. & Adler P. B. Large niche differences emerge at the recruitment stage to stabilize grassland coexistence. Ecol. Monogr. 85, 373–392 (2015).

[b13] Freckleton R. P., Watkinson A. R., Green R. E. & Sutherland W. J. Census error and the detection of density dependence. J. Anim. Ecol. 75, 837–851 (2006). 17009748 10.1111/j.1365-2656.2006.01121.x

[b14] Yenni G., Adler P. B. & Morgan Ernest S. K. Strong self-limitation promotes the persistence of rare species. Ecology 93, 456–461 (2012). 22624200 10.1890/11-1087.1

[b15] Klanderud K. Climate change effects on species interactions in an alpine plant communities. J. Ecol. 93, 127–137 (2005).

[b16] Adler P. B., Leiker J. & Levine J. M. Direct and indirect effects of climate change on a prairie plant community. PLoS ONE 4, e6887 (2009). 19727390 10.1371/journal.pone.0006887 PMC2731204

[b17] Alexander J. M., Diez J. M. & Levine J. M. Novel competitors shape species' responses to climate change. Nature 525, 515–518 (2015). 26374998 10.1038/nature14952

[b18] Ockendon N. . Mechanisms underpinning climatic impacts on natural populations: altered species interactions are more important than direct effects. Glob. Change Biol. 20, 2221–2229 (2014). 10.1111/gcb.12559 24677405

[b19] Adler P. B., Tyburczy W. R. & Lauenroth W. K. Long-term mapped quadrats from Kansas prairie: a unique source of demographic information for herbaceous plants. Ecology 88, 2673 (2007).

[b20] Zachmann L., Moffet C. & Adler P. Mapped quadrats in sagebrush steppe: long-term data for analyzing demographic rates and plant-plant interactions. Ecology 91, 3427 (2010).

[b21] Anderson J., Vermeire L. & Adler P. B. Fourteen years of mapped, permanent quadrats in a northern mixed prairie, USA. Ecology 92, 1703 (2011).

[b22] Anderson J., McClaran M. P. & Adler P. B. Cover and density of semi-desert grassland plants in permanent quadrats mapped from 1915 to 1947. Ecology 93, 1492 (2012).

[b23] Hill R. R. Charting quadrats with a pantograph. Ecology 1, 270–273 (1920).

[b24] Adler P. B., HilleRisLambers J., Kyriakidis P., Guan Q. & Levine J. M. Climate variability has a stabilizing effect on coexistence of prairie grasses. Proc. Natl Acad. Sci. USA 103, 12793–12798 (2006). 16908862 10.1073/pnas.0600599103 PMC1550767

[b25] Lauenroth W. K. & Adler P. B. Demography of perennial grassland plants: survival, life expectancy and life span. J. Ecol. 96, 1023–1032 (2008).

[b26] Chu C. J. . Life form influences survivorship patterns for 109 herbaceous perennials from six semi-arid ecosystems. J. Veg. Sci. 25, 947–954 (2014).

[b27] Fair J., Lauenroth W. K. & Coffin D. P. Demography of *Bouteloua gracilis* in a mixed prairie: analysis of genets and individuals. J. Ecol. 87, 233–243 (1999).

[b28] Ellner S. P. & Rees M. Integral projection models for species with complex demography. Am. Nat. 167, 410–428 (2006). 16673349 10.1086/499438

[b29] Adler P. B., Ellner S. P. & Levine J. M. Coexistence of perennial plants: an embarrassment of niches. Ecol. Lett. 13, 1019–1029 (2010). 20545729 10.1111/j.1461-0248.2010.01496.x

[b30] Merow C. . Advancing population ecology with integral projection models: a practical guide. Methods Ecol. Evol. 5, 99–110 (2014).

[b31] Burnham K. P. & Anderson D. R. Model Selection and Multimodel Inference: A Practical-Theoretical Approach Springer-Verlag (2002).

[b32] Ricker W. E. Stock and recruitment. J. Fish. Res. Board 11, 559–623 (1954).

[b33] Lunn D. J., Thomas A., Best N. & Spiegelhalter D. WinBUGS – a Bayesian modelling framework: concepts, structure, and extensibility. Stat. Comput. 10, 325–337 (2000).

[b34] Brooks S. P. & Gelman A. General methods for monitoring convergence of iterative simulations. J. Comput. Graph. Stat. 7, 434–455 (1998).

[b35] Dalgleish H. J., Moffet C. A., Koons D. N., Hooten M. B. & Adler P. B. Climate influences the population dynamics of three dominant sagebrush steppe plants. Ecology 92, 75–85 (2011). 21560678 10.1890/10-0780.1

[b36] Farrer E. C., Ashton I. W., Knape J. & Suding K. N. Separating direct and indirect effects of global change: a population dynamic modeling approach using readily available field data. Glob. Change Biol. 20, 1238–1250 (2014). 10.1111/gcb.12401 24115317

