# Supplementary material for: Direct effects dominate responses to climate perturbations in grassland plant communities
Source: Nat Commun. 2016 Jun 8;7:11766. doi: 10.1038/ncomms11766 (PMC4899860; doi:10.1038/ncomms11766)
Supplement: Supplementary Information — Supplementary Figures 1-3, Supplementary Tables 1-5 [file ncomms11766-s1.pdf]

Supplementary Figure 1. Comparison of the observed (circles) and predicted mean cover from simulations with an individual-based model either including both climatic covariates and random year effects (squares) or climatic covariates only (triangles). In Figure 2 in the main text, one species from each site (not included here) was presented for demonstration.

Arizona

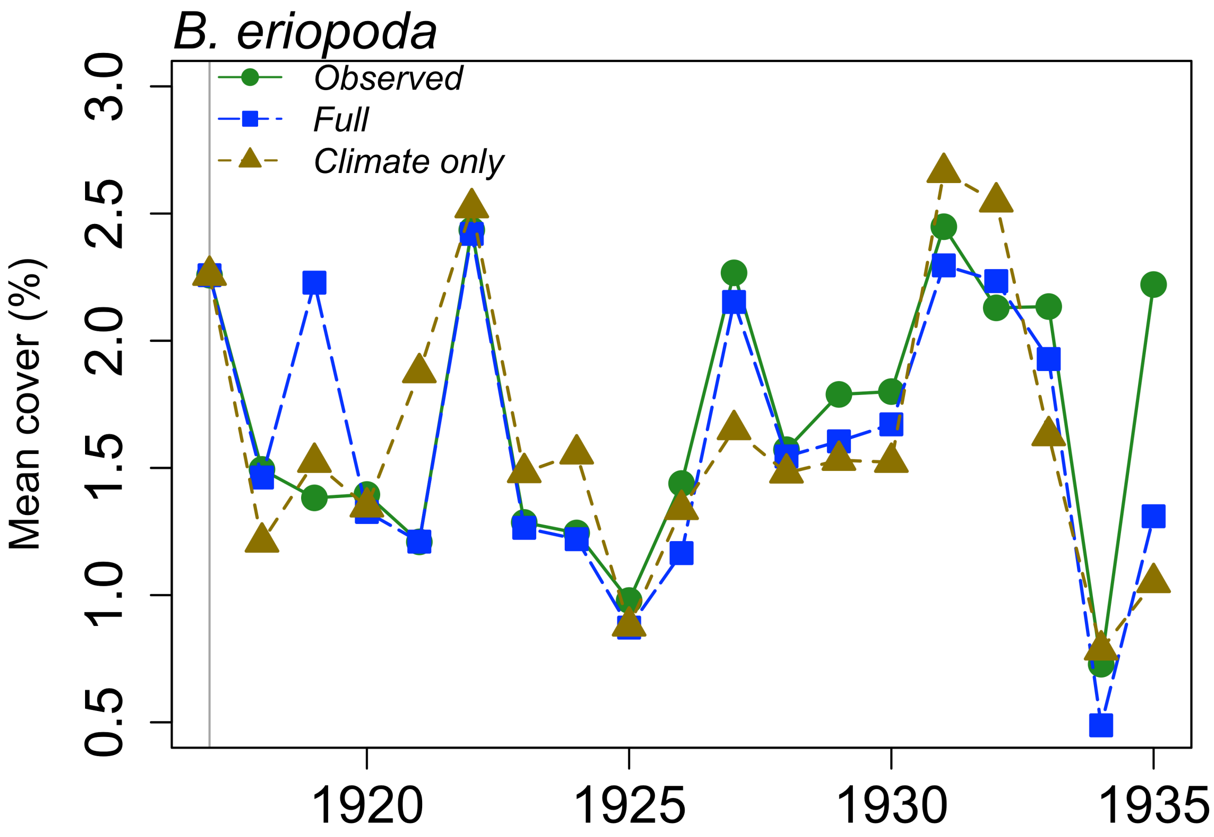

Idaho

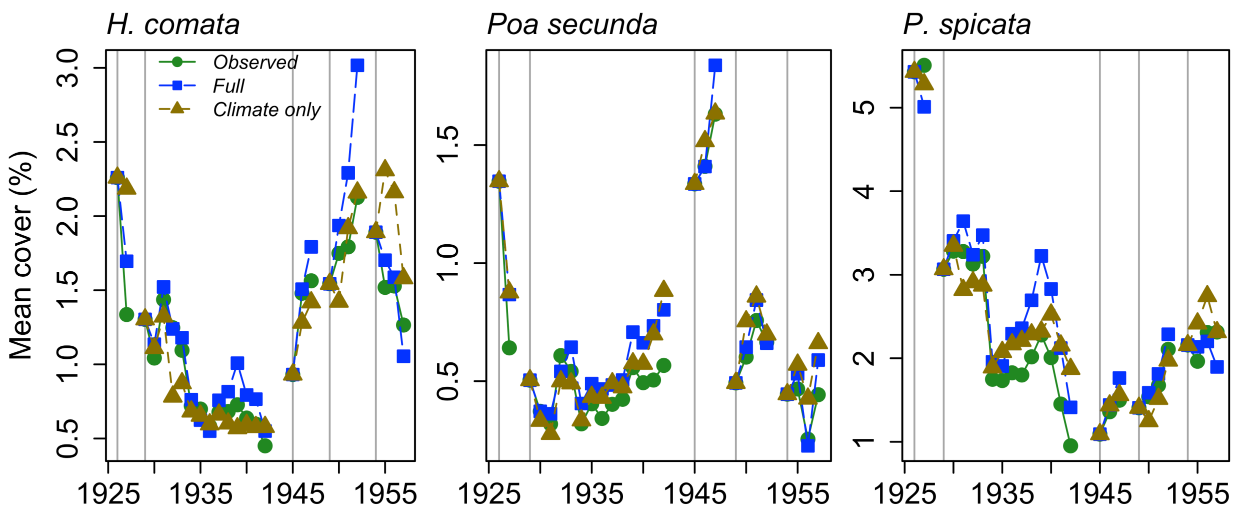

Kansas

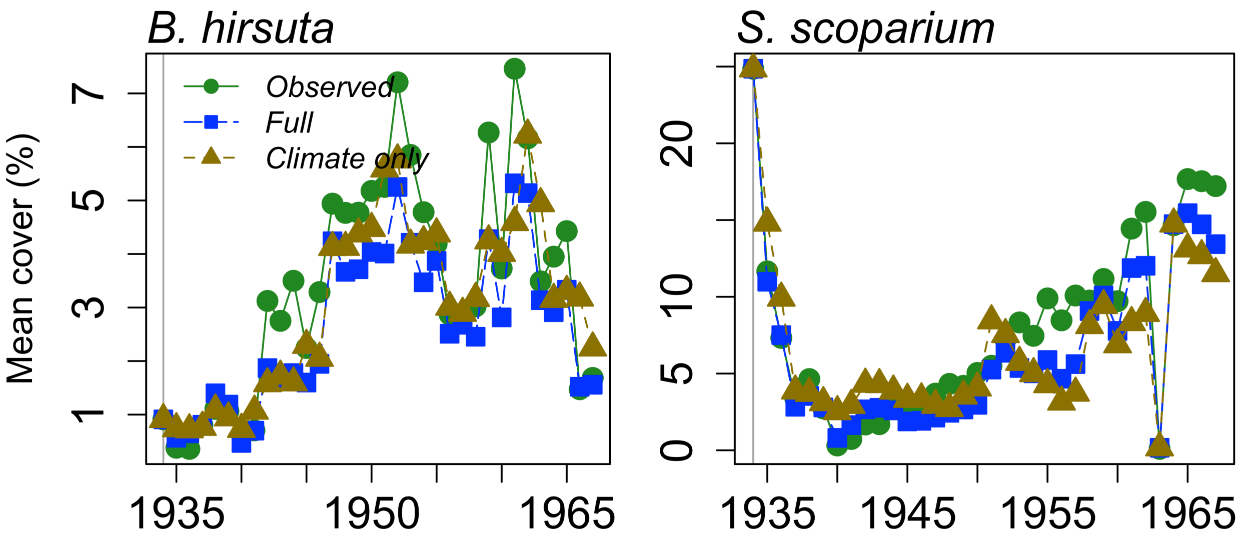

Montana

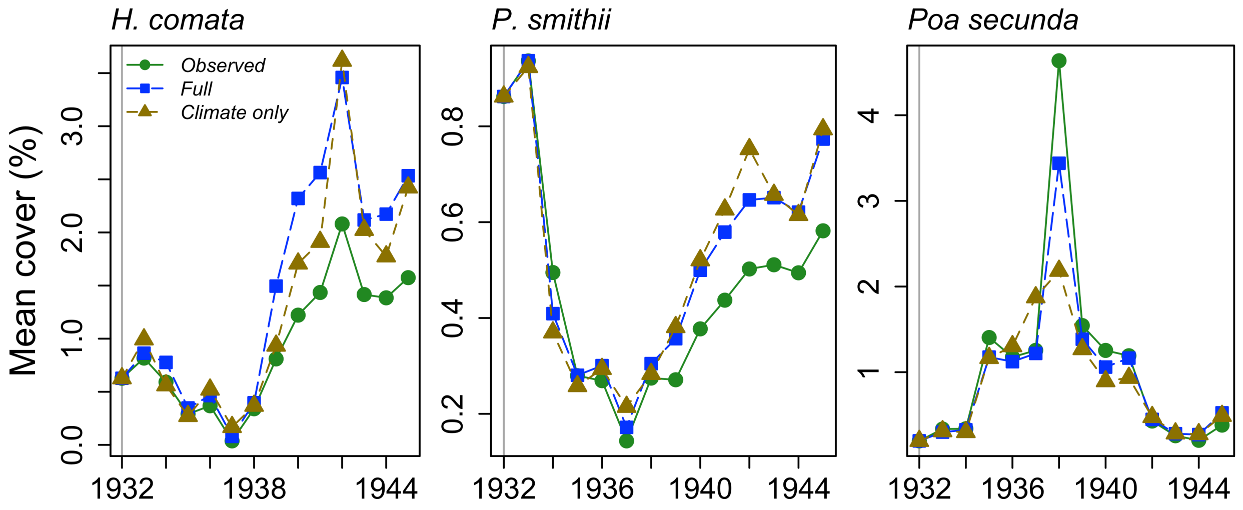

New Mexico

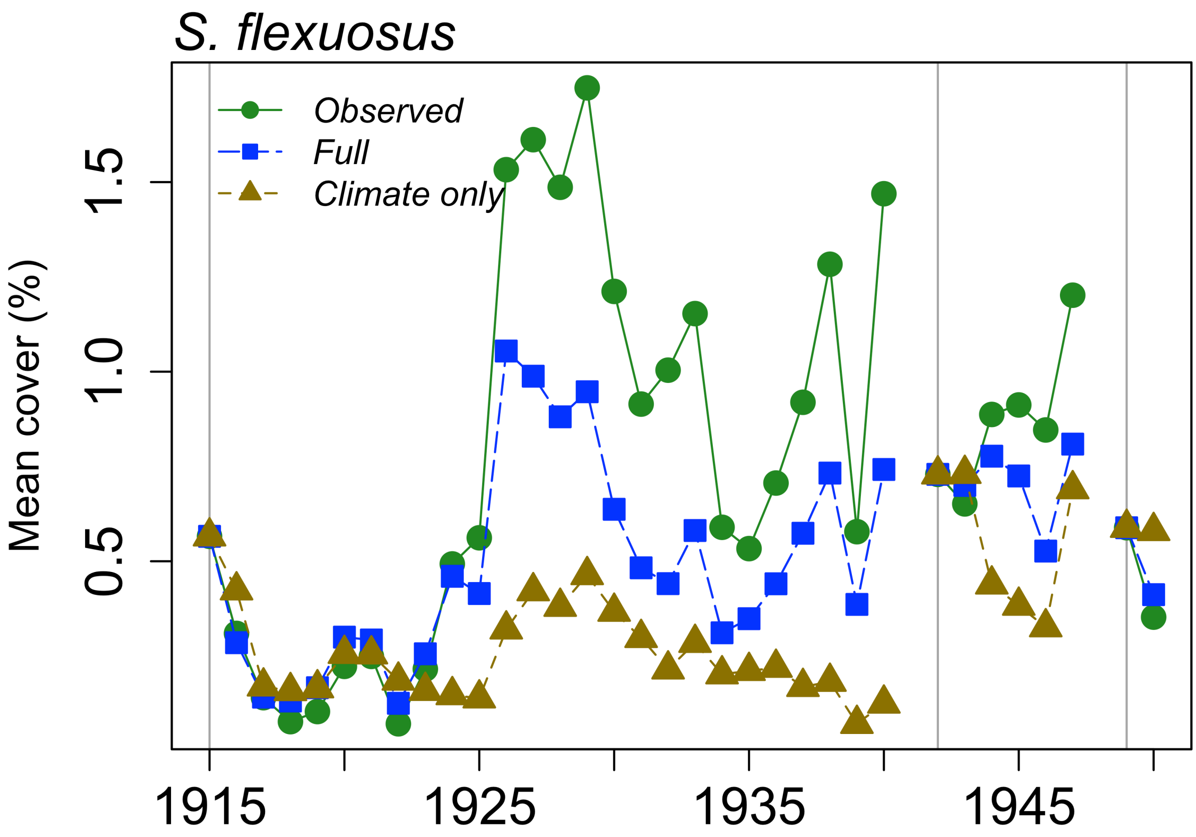

Supplementary Figure 2. Raw full, direct and indirect effects of climate perturbations on equilibrium cover. Climate perturbations included increases in precipitation, temperature, or the variability of precipitation and temperature.

Arizona

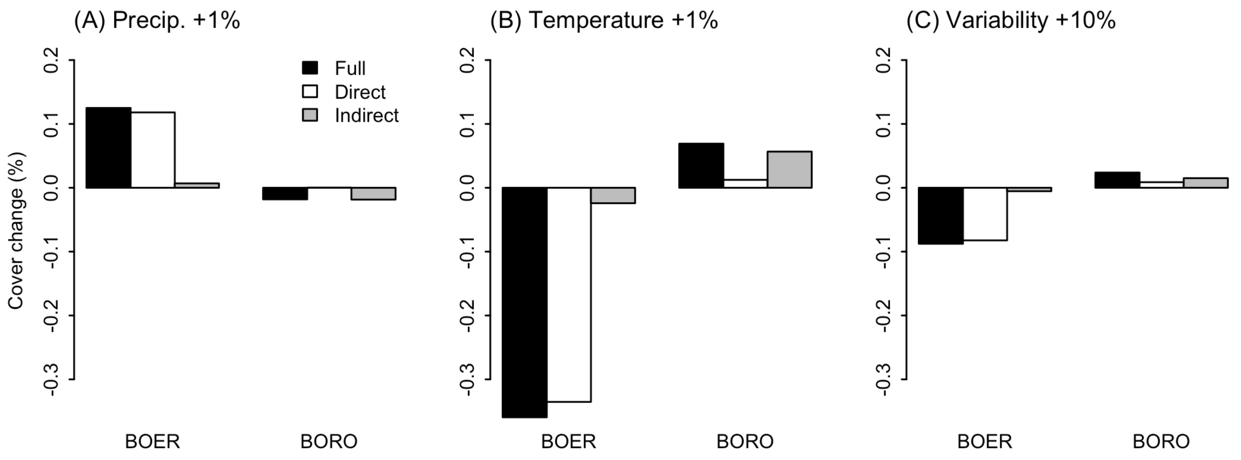

Idaho

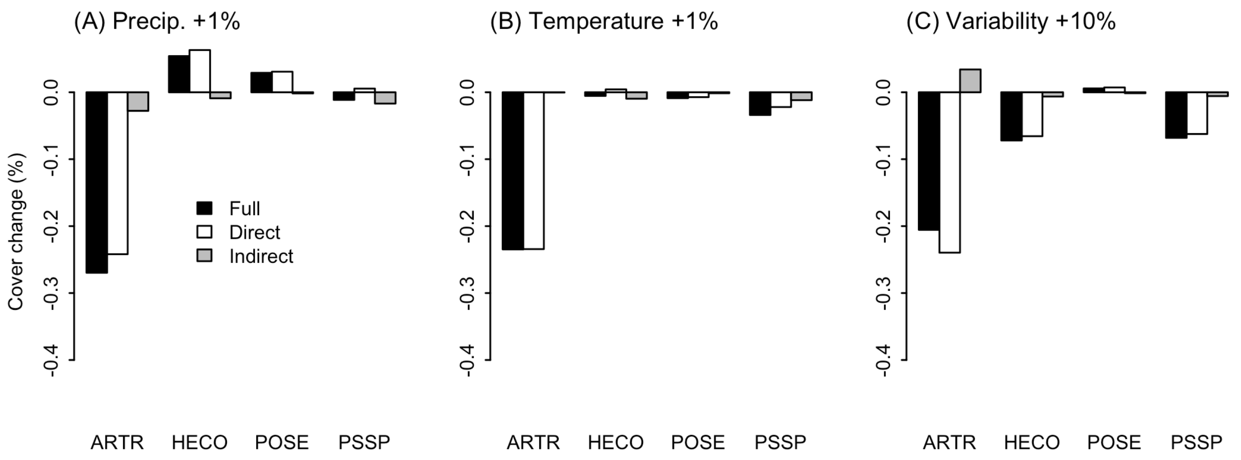

Kansas

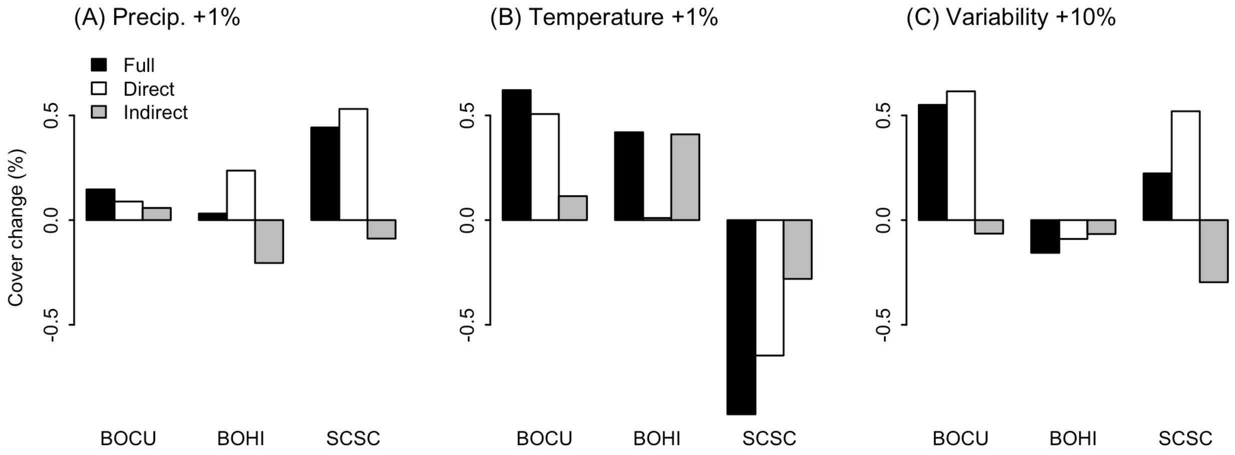

Montana

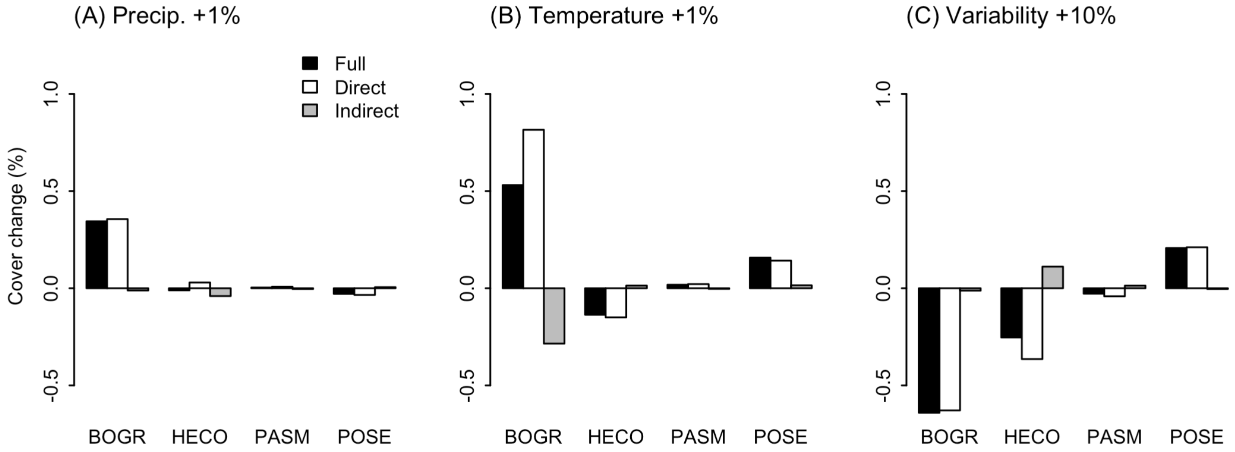

New Mexico

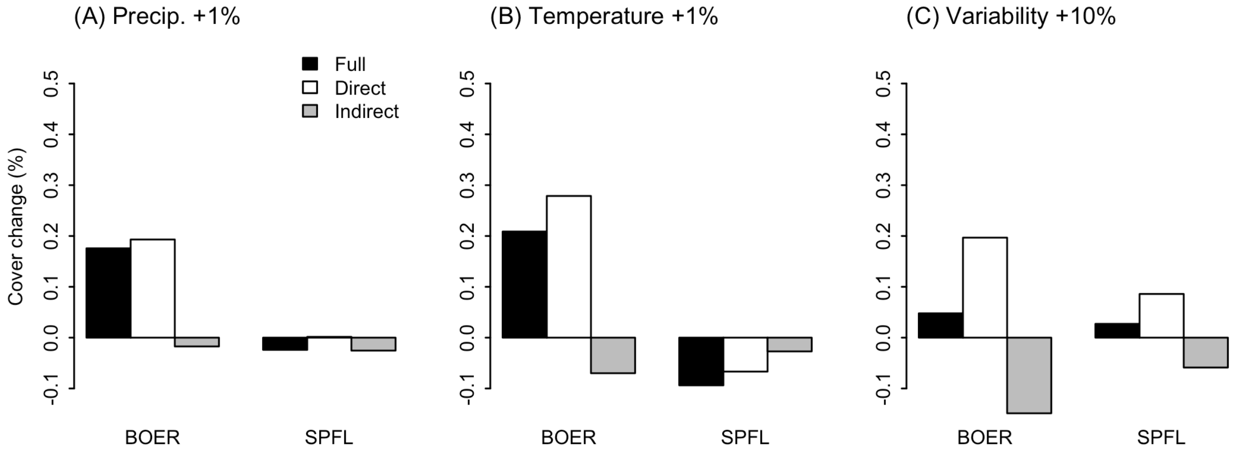

Supplementary Figure 3. Relationships of proportional full effects with proportional direct effects (left panel) and proportional indirect effects (right panel).

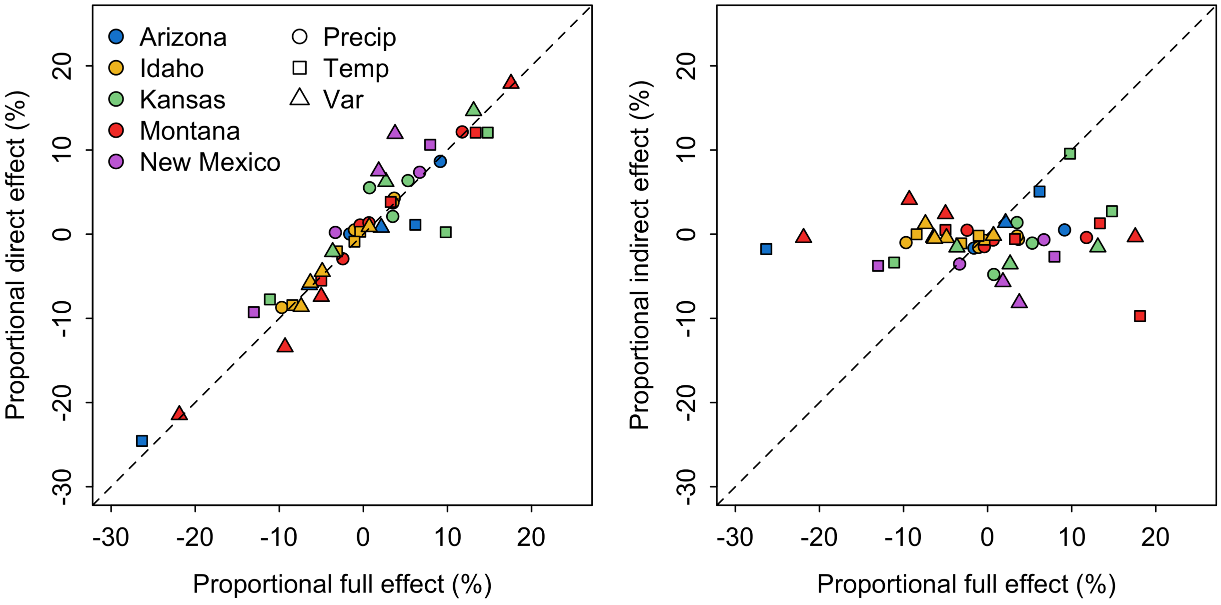

Supplementary Table 1. The proportion of interannual variability in vital rates explained by the climate covariates. The 'Constant model' does not allow any variability in vital rates (neither climate covariates nor random year effects), the 'Climate model' explains temporal variability using climate covariates only, and the 'Full model' explains temporal variability through both climate covariates and random year effects.

Arizona site

	N	Constant model	Climate model	Full model	Contribution of climate covariates *
Growth	*Sum of squared residuals*				
Bouteloua eriopoda	3380	2506.8	2178.1	1986.7	0.63
Bouteloua rothrockii	7962	12509.5	11069.7	9973.2	0.57
Survival	*Residual deviance*				
Bouteloua eriopoda	4538	3148.3	3005.7	2984.5	0.87
Bouteloua rothrockii	14831	16253.7	16001.0	15758.2	0.51
Recruitment	*Residual deviance*				
Bouteloua eriopoda	342	357.7	349.0	293.8	0.14
Bouteloua rothrockii	342	567.4	510.5	368.2	0.29

Idaho site

	N	Constant model	Climate model	Full model	Contribution of climate covariates *
Growth	*Sum of squared residuals*				
Artemisia tripartita	1163	1172.2	1105.9	871.6	0.22
Hesperostipa comata	3092	2038.6	1859.5	1711.0	0.55
Poa secunda	3942	3557.1	3426.2	3239.9	0.41
Pseudoroegneria spicata	5496	3890.8	3750.6	3487.6	0.35
Survival	*Residual deviance*				
Artemisia tripartita	2058	1279.6	1184.2	1174.9	0.91
Hesperostipa comata	4525	3308.8	3145.7	3112.2	0.83
Poa secunda	5893	4983.2	4866.4	4763.9	0.53
Pseudoroegneria spicata	8843	5523.0	5160.1	5066.2	0.79
Recruitment	*Residual deviance*				
Artemisia tripartita	416	537.2	490.0	341.9	0.24
Hesperostipa comata	416	412.7	389.4	274.5	0.17
Poa secunda	416	631.1	569.2	424.0	0.30
Pseudoroegneria spicata	416	742.7	670.7	410.5	0.22

Kansas site

	N	Constant model	Climate model	Full model	Contribution of climate covariates *
Growth	*Sum of squared residuals*				
Bouteloua curtipendula	1089	996.0	884.8	601.5	0.28
Bouteloua hirsuta	812	455.7	416.6	309.4	0.27
Schizachyrium scoparium	601	386.6	336.1	257.1	0.39
Survival	*Residual deviance*				
Bouteloua curtipendula	1710	1596.1	1492.9	1434.6	0.64
Bouteloua hirsuta	1101	779.8	766.7	712.9	0.20
Schizachyrium scoparium	886	690.6	668.9	633.0	0.38
Recruitment	*Residual deviance*				
Bouteloua curtipendula	142	214.5	205.6	129.2	0.10
Bouteloua hirsuta	142	150.5	143.8	115.5	0.19
Schizachyrium scoparium	142	167.4	116.3	105.0	0.82

Montana site

	N	Constant model	Climate model	Full model	Contribution of climate covariates *
Growth	*Sum of squared residuals*				
Bouteloua gracilis	4148	6458.6	3623.6	3458.4	0.94
Hesperostipa comata	1652	2173.7	1803.8	1594.1	0.64
Pascopyrum smithii	6875	2880.8	2738.1	2701.0	0.79
Poa secunda	2537	3444.0	3644.6	2449.0	0.80
Survival	*Residual deviance*				
Bouteloua gracilis	6674	4687.4	4290.3	4266.0	0.94
Hesperostipa comata	2684	2664.4	2361.7	2341.4	0.94
Pascopyrum smithii	9537	8270.9	7753.8	7733.9	0.96
Poa secunda	3839	3402.7	3355.2	3332.1	0.67
Recruitment	*Residual deviance*				
Bouteloua gracilis	215	214.5	195.4	175.4	0.49
Hesperostipa comata	215	281.2	191.4	179.0	0.88
Pascopyrum smithii	215	325.6	282.9	226.5	0.43
Poa secunda	215	271.7	242.2	211.7	0.49

New Mexico site

	N	Constant model	Climate model	Full model	Contribution of climate covariates *
Growth	*Sum of squared residuals*				
Bouteloua eriopoda	11291	6646.7	6333.8	5342.4	0.24
Sporobolus flexuosus	2502	1841.9	1738.9	1565.8	0.37
Survival	*Residual deviance*				
Bouteloua eriopoda	17059	13618.7	13362.1	12929.8	0.37
Sporobolus flexuosus	3927	3461.2	3433.0	3160.3	0.094
Recruitment	*Residual deviance*				
Bouteloua eriopoda	923	1179.6	1078.2	872.3	0.33
Sporobolus flexuosus	923	623.3	552.6	412.4	0.34

*(Climate model – Constant model) / (Full model – Constant model)

Supplementary Table 2. Correlation coefficients for observed and predicted cover from IBM simulations. $\mathbf{Cor_{Obs\ vs.\ Full}}$ represents the correlation coefficients between the observed cover and the predicted cover from the Full models including both random years effects and climate covariates. $\mathbf{Cor_{Obs\ vs.\ Clim}}$ represents the correlation coefficients between the observed cover and the predicted cover from the Climate models including only climate covariates. $\mathbf{Cor_{Obs\ vs.\ Year}}$ represents the correlation coefficients between the observed cover and the predicted cover from the models including only random year effects.

**Study site**	**Species**	$\mathbf{Cor_{Obs\ vs.\ Full}}$	$\mathbf{Cor_{Obs\ vs.\ Clim}}$	$\mathbf{Cor_{Obs\ vs.\ Year}}$
AZ	BOER	0.83	0.70	0.52
AZ	BORO	0.98	0.91	0.80
ID	ARTR	0.94	0.91	0.71
ID	HECO	0.95	0.88	0.89
ID	POSE	0.97	0.93	0.91
ID	PSSP	0.96	0.96	0.85
KS	BOCU	0.94	0.45	0.90
KS	BOHI	0.97	0.88	0.87
KS	SCSC	0.97	0.87	0.81
MT	BOGR	0.92	0.90	0.64
MT	HECO	0.98	0.98	0.67
MT	PASM	0.94	0.90	0.89
MT	POSE	0.99	0.85	0.48
NM	BOER	0.99	0.54	0.77
NM	SPFL	0.90	0.22	0.55

Supplementary Table 3. Values for species' intrinsic per capita growth rates (y-intercept in Fig. 3), equilibrium frequencies (x-intercept in Fig. 3), and the slopes representing the magnitude negative frequency dependence.

**Study site**	**Species**	**Per capita growth rate** (y-intercept)	**Frequency** (x-intercept)	**Negative frequency dependence** (Slope)
Arizona	BOER	0.0937	0.5499	-0.170
Arizona	BORO	1.0230	0.4501	-2.273
Idaho	ARTR	0.0682	0.4513	-0.151
Idaho	HECO	0.278	0.2390	-1.1632
Idaho	POSE	0.437	0.1341	-3.262
Idaho	PSSP	0.192	0.1756	-1.094
Kansas	BOCU	0.742	0.2495	-2.974
Kansas	BOHI	0.469	0.2546	-1.842
Kansas	SCSC	0.415	0.4959	-0.837
Montana	BOGR	0.094	0.3966	-0.238
Montana	HECO	0.681	0.3682	-1.849
Montana	PASM	1.094	0.07563	-14.460
Montana	POSE	0.560	0.1596	-3.510
New Mexico	BOER	0.187	0.7847	-0.238
New Mexico	SPFL	0.460	0.2153	-2.137

Supplementary Table 4. Results for the mixed-effects models with different combinations of variables (NFD and Variance in direct effects) and study sites (the influence of Kansas and Montana sites on the results). Variance represents the variance of direct effects.

Table 4.1. Results for the case excluding the Variance variable

Variable	Estimated value	SE	DF	*t*-value	*P* value
(Intercept)	0.075	0.031	29	2.38	0.0239
NFD	0.0059	0.0035	29	1.69	0.1025

Table 4.2. Results for the case excluding the NFD variable

Variable	Estimated value	SE	DF	*t*-value	*P* value
(Intercept)	0.020	0.021	30	0.957	0.3460
Variance	0.56	0.139	9	4.052	**0.0029**

Following we removed the data from KS and MT individually and together.

Table 4.3. Results for the case removing KS data

Variable	Estimated value	SE	DF	*t*-value	*P* value
(Intercept)	0.025	0.0120	23	2.041	0.0529
NFD	0.0050	0.00232	23	2.170	**0.0406**
Variance	0.38	0.1528	7	2.490	**0.0416**

Table 4.4. Results for the case removing MT data

Variable	Estimated value	SE	DF	*t*-value	*P* value
(Intercept)	0.047	0.0233	20	2.023	0.0566
NFD	0.016	0.0103	20	1.550	0.1368
Variance	0.69	0.138	7	4.995	**0.0016**

Table 4.5. Results for the case removing data from KS and MT

Variable	Estimated value	SE	DF	*t*-value	*P* value
(Intercept)	0.033	0.0177	14	1.889	0.0799
NFD	0.0056	0.00398	14	1.396	0.1843
Variance	0.145	0.454	5	0.318	0.7631

From Table 4.4, if we excluded both KS and MT study sites (more than half our data!), neither NFD nor Variance in direct effects has a significant effect. That we lose the power to detect

significant effects should not be surprising, but it does demonstrate the importance of collecting data across multiple sites.

Supplementary Table 5. Climate covariates selected *a priori* for each study site include three precipitation variables (pptLag, ppt1, and ppt2) and two temperature variables (temp1, temp2). pptLag is the 12 month water-year precipitation in the year preceding an observed year-to-year transition, ppt1 and temp1 are the precipitation and temperature of the critical seasons in the first year of a transition, respectively, and ppt2 and temp2 are the precipitation of the critical seasons in the second year of a transition, respectively. Here 'water year' refers to the period between October 1st of one year and September 30th of the next.

Variables	AZ	KS	ID	MT	NM
pptLag	Water year	Annual	Annual	Water year	Water year
ppt1	Summer	Spring-summer	Fall-spring	Fall-spring	Spring-summer
ppt2	Summer	Spring-summer	Fall-spring	Fall-spring	Spring-Summer
temp1	Summer (Mean)	Spring-summer (Max)	Spring (Mean)	Spring (Mean)	Summer (Mean)
temp2	Summer (Mean)	Spring-summer (Max)	Spring (Mean)	Spring (Mean)	Summer (Mean)
